# A Privacy-Preserving Artificial Intelligence-Driven Sensing System for Distributed Multimodal Risk Detection

**DOI:** 10.3390/s26092864

**Published:** 2026-05-03

**Authors:** Yawen Zhu, Yiwei Song, Yikun Xuan, Yujing Song, Jiahong Pu, Jiehua Li, Manzhou Li

**Affiliations:** 1 China Agricultural University, Beijing 100083, China; 2 National School of Development, Peking University, Beijing 100871, China

**Keywords:** artificial intelligence-driven sensing, federated learning, multimodal sensing, privacy-preserving risk detection, cross-modal semantic reasoning

## Abstract

Withthe widespread deployment of intelligent terminals, mobile payment platforms, and Internet of Things devices, security systems are being progressively transformed from traditional transaction outcome analysis toward an intelligent perception paradigm centered on user behavior, device states, and environmental context. To address the challenges of multimodal data heterogeneity, non-independent and identically distributed data across nodes, and the difficulty of centralized modeling under privacy constraints in distributed scenarios, an artificial intelligence-driven federated multimodal security perception framework, namely FMS-LLM, is proposed. At its core, the framework introduces a Non-IID adaptive federated fusion mechanism that achieves dual-level alignment—structural alignment via parameter-level masks and semantic alignment via feature consistency constraints—to effectively mitigate cross-node distribution discrepancies. Additionally, an LLM-driven semantic enhancement module is developed, utilizing trend-guided token selection and inertia-suppression to map low-level sensing features into high-level risk semantic representations, thereby supporting logical reasoning and explainable decision-making. This framework takes user behavioral sensing data, device state information, environmental context data, and transaction behavior data as inputs, and constructs an integrated security analysis pipeline of “perception–collaboration–reasoning”. Experimental results on the distributed multimodal security perception task demonstrate that the proposed method achieves an Accuracy of 91.62%, a Precision of 91.04%, a Recall of 90.37%, an F1-score of 90.70%, and a ROC-AUC of 94.73%, consistently outperforming baseline methods including Logistic Regression, Random Forest, LSTM, the centralized multimodal deep model, FedAvg, FedProx, and MOON. Under strongly Non-IID conditions, when α=0.1, the model still maintains an Accuracy of 88.47% and an F1-score of 87.11%, demonstrating stronger cross-node robustness. The ablation study further indicates that the complete model attains the best classification performance while reducing communication cost to 18.92 MB/Round. These results demonstrate that the proposed method can effectively fuse multi-source sensing information under privacy-preserving conditions and support intelligent security perception tasks with higher accuracy, stronger robustness, and improved interpretability.

## 1. Introduction

With the rapid development of intelligent terminals, mobile payments, and the Internet of Things, financial services are evolving from traditional counter- and web-based models toward real-time, scenario-driven, and intelligent operations [1]. Accordingly, financial security is shifting from post-hoc analysis based on transaction records and rule-based auditing to multimodal, real-time perception and risk decision-making that incorporate user behavior, device states, and contextual information [2]. In scenarios such as remote account opening, mobile payments, and digital lending, efficiently integrating distributed multi-source data and extracting latent risk patterns under strict privacy constraints has become a critical challenge [3]. In this context, distributed intelligent approaches, particularly federated learning, have emerged as promising solutions with significant theoretical and practical value [4].

Existing studies show that traditional financial security methods are primarily based on centralized data analysis and rule-driven modeling paradigms [5], including expert-defined threshold-based approaches and statistical or shallow machine learning methods with handcrafted features (e.g., logistic regression, support vector machines, and decision trees) [6]. These approaches, characterized by simplicity and interpretability, have played a significant role in early financial risk control and achieved reasonable performance on structured transaction data [7]. However, as fraudulent activities become increasingly covert, dynamic, and complex, their limitations have become more apparent [8]. On one hand, they mainly rely on transaction data and fail to fully leverage multimodal information from terminal devices [9], user interactions, and contextual environments, resulting in limited risk perception [10]. On the other hand, their dependence on centralized data conflicts with real-world constraints such as privacy protection [11], regulatory compliance, and commercial boundaries, leading to data silos [12]. Furthermore, they struggle to handle pronounced Non-IID data across devices, users, and scenarios, which weakens their generalization capability [13]. More importantly, their limited capacity to model complex relationships among heterogeneous multimodal data restricts their ability to capture the coupling between behavior, device, environment, and transactions, thereby reducing their effectiveness in detecting advanced threats such as account takeover and distributed fraud.

In recent years, the rapid advancement of deep learning has driven financial security modeling from shallow, handcrafted feature-based approaches toward automatic representation learning [14]. Models such as convolutional neural networks (CNN), recurrent neural networks (RNN), long short-term memory (LSTM), and Transformers can extract discriminative features from high-dimensional, complex, and noisy data [15]. In multimodal scenarios, deep learning captures behavioral evolution through sequence modeling, highlights critical risk cues via attention mechanisms, and integrates user behavior, device states, and contextual information through multimodal fusion [16], thereby significantly improving the accuracy and robustness of risk detection [17]. Meanwhile, federated learning, through its paradigm of keeping data local while collaboratively optimizing models, provides essential support for privacy-sensitive, cross-device, and cross-institutional modeling [18]. However, in real-world multimodal financial scenarios, conventional federated learning methods face significant hurdles. While advanced variants like FedProx [19] and MOON [20] attempt to mitigate Non-IID challenges, they exhibit inherent limitations when applied to multi-source financial data. FedProx introduces a proximal regularization term to stabilize local updates, yet its coarse-grained constraint treats all model parameters uniformly, failing to account for the heterogeneous importance and feature-level misalignment of distinct modalities across different nodes. Similarly, MOON utilizes model-contrastive learning to enhance representation consistency, but it primarily focuses on a single representation space and lacks the capacity to capture complex cross-modal semantic coupling or address the semantic drift between high-dimensional sensing signals and structured transaction logs. These deficiencies often lead to sub-optimal convergence and limited generalization in highly heterogeneous environments [21]. Therefore, effectively integrating federated learning with fine-grained multimodal alignment and high-level semantic reasoning has become a key research direction [22]. Empirical studies further support this trend, such as MFFER [23], and federated cross-modal dynamic networks [24], which demonstrate the potential of sophisticated collaborative frameworks. Traditional risk models, including ARCH/GARCH variants and standard Value-at-Risk frameworks, capture temporal volatility clustering with considerable success, yet they remain fundamentally univariate and are difficult to integrate into high-dimensional learning pipelines [25,26]. More importantly, neither classical econometric models nor most recent deep learning approaches adequately address the joint task of modeling the full return distribution, rather than only its conditional mean and a limited subset of risk characteristics. In recent years, machine learning has substantially advanced empirical asset pricing, with tree-based models and deep neural networks showing clear gains over linear benchmarks in out-of-sample return prediction [27]. Related progress has also been achieved by embedding no-arbitrage structure into deep architectures [28], by exploiting temporal dependence through LSTM networks [29], and by modeling long-range cross-sectional and sequential interactions via Transformer-based attention mechanisms [30,31]. However, despite these methodological advances, existing deep learning models for asset pricing still largely follow a point-prediction paradigm, thereby discarding substantial economically meaningful information.

Motivated by the above analysis, we propose FMS-LLM, a distributed multimodal financial security perception framework that establishes a synergistic optimization paradigm between federated learning and cross-modal semantic reasoning. Rather than a simple assembly of independent components, our framework introduces a tightly coupled architecture designed to reconcile the intrinsic conflicts between cross-node data heterogeneity and cross-modal alignment through a well-designed technical integration. The framework is characterized by an adaptive federated fusion mechanism that mitigates Non-IID noise by quantifying distribution discrepancies, coupled with a semantic enhancement module where the large language model acts as an integrated auxiliary supervisor. By enforcing a closed-loop consistency constraint between high-level risk rationales and low-level perceptual features, the proposed method addresses the challenge of semantic drift in decentralized environments. This methodological coupling enables robust and interpretable risk identification driven by multi-source sensing information, providing a technically grounded solution for privacy-sensitive and heterogeneous real-world financial scenarios.

The main contributions of this study are summarized as follows.

1. To address the challenge of limited risk perception in traditional transaction-based systems, a distributed multimodal sensing architecture for financial security is constructed, where user behavioral sensing data, device state information, environmental context, and transaction data are jointly modeled to provide a comprehensive technical pipeline for multimodal intelligent perception;
2. To overcome the performance degradation caused by data silos and strong Non-IID characteristics across nodes, a Non-IID-aware federated multimodal fusion mechanism is designed, incorporating dynamic weighting, modality alignment, and adaptive aggregation strategies to effectively mitigate distribution discrepancies across terminals;
3. In response to the high communication overhead and deployment difficulties in large-scale distributed environments, a communication-efficient federated compression and update strategy is developed, employing model pruning, gradient compression, and local update mechanisms to enhance system deployability;
4. To mitigate the lack of interpretability in deep learning models for auditing and regulatory requirements, a large language model-driven cross-modal semantic enhancement and risk reasoning module is introduced, mapping low-level sensing features to high-level risk semantic representations to improve knowledge integration and decision support;
5. Finally, to provide empirical validation for the proposed framework under complex financial scenarios, comprehensive evaluations are conducted on real or simulated distributed multimodal financial sensing tasks, demonstrating the effectiveness of the integration in terms of classification performance, Non-IID robustness, communication efficiency, and interpretability.

In summary, the proposed framework integrates distributed sensing, federated collaboration, multimodal fusion, and large language model (LLM)-based semantic reasoning into a unified architecture, establishing a complete technical pipeline from local terminal perception to global risk interpretation. Compared with traditional centralized approaches, greater emphasis is placed on privacy preservation and cross-node collaboration; compared with conventional deep learning methods, the framework extends beyond feature extraction and classification to address robust learning and semantic reasoning under complex distribution conditions; and compared with standard federated learning methods, it is more suitable for intelligent security perception tasks involving heterogeneous multimodal financial data. Therefore, this study provides both a novel theoretical framework for distributed financial security modeling and a practical reference for building more intelligent, trustworthy, and efficient financial risk control systems.

## 2. Related Work

### 2.1. Sensor-Based Intelligent Financial Security Perception Methods

Sensor-based financial security methods model multimodal sensing signals, including user behavior, device states, and environmental context, extending risk perception from traditional post-hoc transaction analysis to real-time monitoring of interaction processes and terminal conditions [32], thereby enhancing anomaly detection and early warning capabilities [33]. Early approaches primarily relied on single-modality modeling, such as using touch behavior for continuous authentication, device fingerprints for trust assessment [34], or spatiotemporal information for transaction validation, which partially overcame the limitations of relying solely on structured transaction data [35]. However, these methods implicitly assume that a single modality is sufficient for risk identification [36], whereas in practice financial risks often emerge from the aggregation of multiple weak anomalies across behavioral, device, and environmental dimensions [37]. As a result, single-modality approaches struggle to capture complex risk patterns and are sensitive to noise, missing data, and environmental variations, leading to limited robustness and increased false positives and false negatives [38]. With the rapid growth of mobile finance and distributed terminal access, these limitations become more pronounced [39]. Therefore, developing multimodal financial security perception frameworks that jointly model behavioral, device, environmental, and transactional data to extract risk cues from heterogeneous sources has become a critical direction for improving accuracy, robustness, and cross-scenario adaptability [40].

### 2.2. Federated Learning in Distributed Intelligent Systems

Federated learning, by keeping data local while enabling collaborative model optimization [41], provides a critical solution for privacy-preserving and highly distributed financial security modeling, effectively avoiding the privacy and compliance risks associated with centralized data aggregation [42]. In this paradigm, individual devices or institutions train models on local data and share model updates to achieve global coordination, supporting cross-device, cross-platform, and cross-institutional risk modeling [43]. However, classical parameter-averaging approaches face multiple challenges in financial security scenarios [44]. First, data across terminals are highly heterogeneous and exhibit strong Non-IID characteristics, causing slow convergence, unstable training, and degraded generalization when using simple aggregation strategies [45]. To address these challenges, several advanced federated learning variants have been proposed, such as FedMA, FedPer, and FedBN. FedMA employs Bayesian non-parametric matching to align neurons across local models, thereby accommodating structural heterogeneity more effectively than coordinate-wise averaging [46]. Meanwhile, personalization-based approaches such as FedPer and FedBN focus on mitigating distribution shifts; FedPer partitions the network into shared base layers and local personalization layers to capture client-specific patterns [47], while FedBN retains local batch normalization layers to handle feature shifts across different domains [48]. While these methods significantly enhance robustness under Non-IID conditions, they primarily target single-modality architectures and remain less effective in addressing the complex inter-modal misalignments and semantic inconsistencies inherent in distributed multimodal financial sensing. Second, multimodal data further amplify this heterogeneity due to differences in modality types, sampling frequencies, completeness, and information quality, making cross-modal collaboration and effective fusion more difficult [49]. Additionally, practical deployment must consider communication efficiency and system security: large models and frequent communication introduce significant overhead [50], while open federated environments are vulnerable to model poisoning, anomalous updates, and backdoor attacks [51]. Although existing studies have made progress in Non-IID optimization, model compression, and robust aggregation, they mainly focus on single-modality tasks and pay limited attention to multimodal collaboration, feature alignment, and complex heterogeneous environments [52]. Therefore, developing specialized federated learning frameworks tailored to multimodal financial security scenarios—capable of balancing privacy preservation, communication efficiency, robustness, and global generalization—has become a critical research challenge [53]. Existing studies generally distinguish between two types of uncertainty in predictive models: aleatoric uncertainty, which refers to the inherent randomness in the data-generating process and cannot be eliminated by increasing data or improving the model, and epistemic uncertainty, which arises from parameter uncertainty and structural errors caused by limited samples and is, in principle, reduced as the sample size increases [54]. If these two sources of uncertainty are not differentiated or are entirely ignored, robust risk signaling becomes difficult to achieve. Bayesian neural networks provide a theoretical framework for uncertainty modeling by imposing prior distributions over model weights and estimating posterior distributions through Bayesian inference [55]; however, Bayesian inference in large-scale neural networks remains computationally expensive, and existing applications are still largely confined to relatively small-scale settings [54]. In addition, although certain progress has been made in high-dimensional financial forecasting and probabilistic prediction frameworks [27,56], unified uncertainty modeling for high-dimensional and complex tasks remains insufficiently explored.

### 2.3. Large Language Models for Multimodal Understanding and Reasoning

The introduction of LLMs marks a paradigm shift in financial security from feature-based perception to semantic understanding and reasoning [57,58]. Benefiting from large-scale pretraining, LLMs possess strong capabilities in contextual modeling, knowledge retention, and logical reasoning [59,60], enabling them not only to process natural language but also to serve as semantic hubs for integrating heterogeneous multimodal inputs, including user behavior, device states, environmental context, and transaction data [61,62]. Currently, LLMs have demonstrated significant practical value in diverse financial risk control scenarios. For instance, they are extensively utilized in fraud text detection to identify deceptive linguistic patterns or social engineering tactics in customer communications, and in automated financial report analysis to scrutinize textual disclosures for potential accounting inconsistencies or hidden credit risks. In financial security tasks, this allows systems to go beyond binary risk detection and generate high-level semantic explanations regarding anomaly origins [63], behavioral deviations, and potential attack patterns, thereby better supporting auditing, regulatory compliance, and decision-making [64]. The prevailing approach follows a “low-level perception + high-level semantic reasoning” paradigm [65], where multimodal encoders first transform raw sensory and structured data into intermediate representations, which are then processed by LLMs under task-specific context, rules, and prior knowledge to produce interpretable outputs [66]. However, several challenges remain: LLMs have limited capability in directly modeling raw temporal sensor data and thus rely on dedicated perception modules [67]; their large scale incurs substantial computational costs, making deployment on distributed terminals impractical [68]; and improper handling of intermediate multimodal representations may introduce additional privacy risks [69]. Therefore, a more practical approach is to position LLMs as back-end semantic enhancement and reasoning modules [70], jointly designed with federated, multimodal perception frameworks to achieve both strong performance and interpretability under privacy constraints [71].

## 3. Materials and Method

### 3.1. Data Collection

A dataset was constructed in this study for the task of distributed multimodal financial security perception, covering the period from 1 January 2023 to 31 December 2024, in order to ensure sufficient representativeness with respect to seasonal variation, holiday fluctuations, terminal replacement, and the evolution of user behavior, as shown in Table 1. The data were primarily collected from the Android mobile terminal platform, mobile payment and digital finance application testing platforms, built-in terminal sensor interfaces, and application-side business log systems, and were further integrated with a semi-realistic business simulation environment to achieve unified collection and temporal alignment. User behavior data were acquired from the Android terminal interaction logging platform, including fine-grained interaction information such as touch trajectories, click intervals, sliding speed, page dwell time, input rhythm, and interface switching paths. The collection platforms consisted of mobile payment application testing platforms and digital account management applications deployed on terminals running Android 12 to Android 14. The recording process was implemented through the combination of system-level event monitoring and application-side frontend logging, with temporal precision reaching the millisecond level.

Device sensing data were obtained from built-in accelerometers, gyroscopes, and device state monitoring interfaces, through which device posture variation, motion states, and hardware operating characteristics during interaction processes were synchronously collected via the Android Sensor API and terminal state acquisition services. The sampling schedule was strictly aligned with user operation windows and was continuously recorded throughout the two-year period. High-frequency continuous sampling was adopted during high-risk operation stages, whereas periodic sampling was employed during routine usage stages, so that dynamic sensitivity and data scale control could be jointly maintained. Environmental perception data were mainly collected from Android system context interfaces, the Amap location service, and network state monitoring modules. The recorded information included precise timestamps, city-level geographic locations, network type switching, IP environment variation, device connection states, and system runtime environments. These data were acquired through the combination of fixed-interval polling and event-triggered collection, thereby enabling relatively complete characterization of the external environmental conditions.

Transaction behavior data were collected from the mobile payment application testing platform and the digital finance business log platform during the same time period. These data mainly recorded structured business information such as transaction amount, transaction type, transaction frequency, operation path, session duration, and abnormal operation labels. To ensure the reliability of the ground truth, the data annotation process followed a rigorous three-stage verification pipeline: initial automated labeling based on preset business rules, detailed manual review by financial security experts, and a final consensus-based audit to resolve discrepancies. Specifically, annotation rules were established using logic-based triggers, where samples were flagged as anomalous if they exhibited severe deviations from historical user profiles, such as abrupt geographic shifts combined with abnormal input rhythms or suspicious device fingerprint variations. Within the final collection of 60,000 transaction records, 51,240 samples were identified as normal while 8760 were categorized as anomalous, resulting in a ratio of approximately 5.8:1, which provides a representative distribution for evaluating risk perception robustness.

The overall dataset was organized on a per-terminal basis, where different devices corresponded to different local nodes, thereby forming a distributed heterogeneous data structure consistent with the federated learning setting and providing a relatively realistic data foundation for subsequent multimodal fusion, Non-IID modeling, and risk reasoning.

### 3.2. Data Preprocessing and Augmentation Strategy

To address inconsistencies in temporal resolution, noise distribution, and feature space across modalities, a unified preprocessing pipeline is designed, including temporal alignment, denoising, normalization and encoding, and data augmentation. Multimodal data are mapped onto a unified temporal grid T. For continuous signals, linear interpolation is applied:

(1) $${\hat{x}}^{\left(m\right)}\left(\tau \right)=x_{i}^{\left(m\right)}+\frac{\tau -t_{i}^{\left(m\right)}}{t_{i+1}^{\left(m\right)}-t_{i}^{\left(m\right)}}\left(x_{i+1}^{\left(m\right)}-x_{i}^{\left(m\right)}\right),$$

where $t_{i}^{\left(m\right)}$ and $x_{i}^{\left(m\right)}$ denote the timestamp and observation of the *i*-th sample in modality *m*. A missing mask $M^{\left(m\right)}\left(\tau_{k}\right)$ is introduced as:

(2) $$M^{\left(m\right)}\left(\tau_{k}\right)=\left\{\begin{array}{cc} 1, & \mathrm{if}\;\mathrm{observations}\;\mathrm{exist},\\ 0, & \mathrm{otherwise}, \end{array}\right.$$

to distinguish missing data from zero values. Statistical methods are used to identify noise anomalies. The standardized deviation $z_{i}$ is defined as:

(3) $$z_{i}=\frac{x_{i}-\mu_{w}}{\sigma_{w}+\epsilon },$$

where $\mu_{w}$ and $\sigma_{w}$ are the mean and standard deviation within a sliding window. A robust metric $r_{i}$ based on median absolute deviation is also adopted:

(4) $$r_{i}=\frac{|x_{i}-\tilde{x}|}{\mathrm{MAD}+\epsilon },$$

where $\tilde{x}$ is the median and MAD denotes the median absolute deviation. Detected noise points are smoothed using a moving average:

(5) $${\bar{x}}_{i}=\frac{1}{2K+1}\sum_{j=i-K}^{i+K}x_{j}.$$

Continuous features are standardized as:

(6) $$x^{\prime }=\frac{x-\mu }{\sigma +\epsilon },$$

where μ and σ are computed from the training set. Categorical variables $v_{c}$ are embedded into dense representations:

(7) $$v_{c}=W_{e}e_{c},$$

where $e_{c}$ is the one-hot vector and $W_{e}$ is the embedding matrix. Temporal features are encoded using sinusoidal functions:

(8) $$\phi \left(t\right)=\left[\sin\left(\frac{2\pi t}{P}\right),\cos\left(\frac{2\pi t}{P}\right)\right],$$

where *P* denotes the period. To improve robustness, several perturbation strategies are introduced. Temporal jitter:

(9) $${\tilde{t}}_{i}=t_{i}+\delta_{i},\;\delta_{i}∼U\left(-\Delta ,\Delta \right),$$

where $\delta_{i}$ denotes a random temporal offset. Behavioral perturbation:

(10) $$\tilde{x}=x+\eta ,\;\eta ∼N\left(0,\sigma^{2}I\right),$$

where x represents the original behavioral feature vector, η is Gaussian noise, $\sigma^{2}$ controls the perturbation intensity, and I is the identity matrix. Modality-missing simulation:

(11) $${\tilde{h}}^{\left(m\right)}=q^{\left(m\right)}h^{\left(m\right)},\;q^{\left(m\right)}∼\mathrm{Bernoulli}\left(p_{m}\right),$$

where $h^{\left(m\right)}$ denotes the feature representation of modality *m*, and $q^{\left(m\right)}$ is a Bernoulli random variable indicating whether the modality is retained ($q^{\left(m\right)}=1$) or masked ($q^{\left(m\right)}=0$), with $p_{m}$ being the retention probability. In our experimental implementation, $p_{m}$ is varied within the range of 0.5 to 1.0 during the training phase to simulate different levels of modality incompleteness, thereby ensuring that the framework maintains high robustness and generalization capability across diverse terminal environments where data may be partially unavailable. Overall, this pipeline enhances data consistency, suppresses noise, and improves model robustness under multimodal and incomplete-input scenarios.

### 3.3. Proposed Method

#### 3.3.1. Overall

At the model design level, the proposed FMS-LLM framework is constructed along the main line of “local perception encoding–federated collaborative optimization–semantic-enhanced reasoning.” First, the multimodal inputs after unified representation are fed into their corresponding local encoding branches. Among them, behavioral sequences are processed by a temporal encoding network to extract dynamic interaction features; device sensing signals are processed by a lightweight perception encoder to derive motion and state representations; and environment- and transaction-related features are transformed into unified embedding representations through a structured mapping layer. Subsequently, features from different modalities are passed into an intra-node multimodal fusion module, where cross-modal alignment is achieved through attention weighting and projection into a shared semantic space, thereby producing a node-level fused representation. Initial risk discrimination is then performed under the constraint of a local classification head. Thereafter, local model parameters and compact representations are uploaded to the federated server. On the server side, aggregation is not performed through simple averaging; instead, adaptive aggregation weights are assigned according to inter-node distribution discrepancy, local training stability, and modality completeness, thereby producing a more robust global model. The aggregated parameters are then redistributed to all terminals for subsequent iterations, such that local feature learning and global consistency constraints are reinforced in an alternating manner. During this process, the communication-efficient module operates synchronously along the parameter update path, reducing transmission burden while preserving convergence performance through sparse uploading, compressed updates, and local multi-step optimization. Finally, the global fused representations produced at the federated stage are fed into the LLM-based semantic reasoning module, where they are first transformed into structured risk description units and then combined with predefined prompt templates to complete cross-modal semantic mapping, risk level determination, and rationale generation. In this way, a complete closed loop is established, spanning low-level sensing signal representation, cross-node collaborative modeling, and high-level interpretable risk reasoning. All modules are tightly connected in a coherent manner and jointly support the distributed multimodal security perception task.

#### 3.3.2. Non-IID Adaptive Federated Multimodal Fusion Mechanism

Within the proposed framework, the Non-IID adaptive federated multimodal fusion mechanism is developed around three interconnected levels, namely node discrepancy modeling, modality-consistent alignment, and dynamic aggregation optimization. Its implementation is tightly coupled with the processes of submodel uploading, server-side reconstruction, and mask generation.

**Figure 1:**
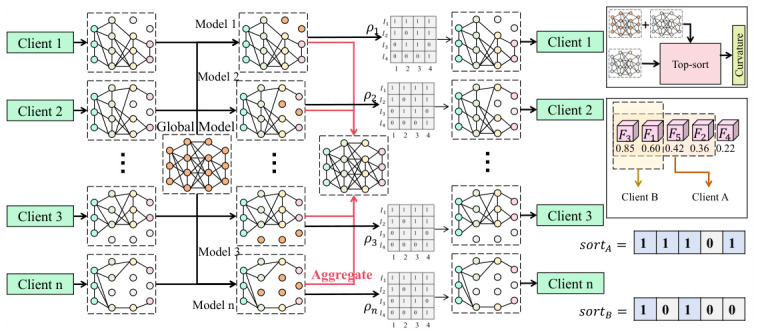
Schematic illustration of the Non-IID adaptive federated multimodal fusion mechanism.

As shown in Figure 1, for each client node, the local model consists of a shared backbone encoder and modality-specific branches, where the shared parameters are denoted by $\theta^{sh}$ and the modality-branch parameters are denoted by $\theta_{k}^{m}$. Features from different modalities are first mapped into a unified embedding space to form $f_{k}^{m}$, after which attention-based fusion is performed to obtain the node-level representation $f_{k}=\sum_{m}\alpha_{k}^{m}f_{k}^{m}$, where the weights $\alpha_{k}^{m}$ are generated by a learnable gating network and are used to characterize the importance of different modalities at the current node. Under Non-IID settings, the data distributions $P_{k}\left(x\right)$ across nodes exhibit significant deviation. Therefore, on the server side, the uploaded submodels are first structurally reconstructed by partitioning the global model parameters into multiple subspaces $\left\{\theta^{\left(i\right)}\right\}$, and a mask matrix $m_{k}$ is generated through importance estimation. In essence, this mask performs sparse selection in the parameter space and satisfies $\theta_{k}=m_{k}⊙\theta$, thereby enabling different clients to participate only in the updates of the most relevant submodels. The mask is generated according to parameter sensitivity jointly measured by local gradients and curvature information. Specifically, a score is constructed through approximate second-order information as $S_{i,k}\approx \left|\nabla L_{k}\left(\theta^{\left(i\right)}\right)\cdot H^{-1}\right|$, followed by ranking and truncation, thereby realizing the adaptive mechanism illustrated in the figure, in which different clients retain different proportions of parameters. During aggregation, simple averaging is abandoned and a distribution-aware weight $w_{k}=\frac{1}{1+\mathrm{MMD}(P_{k},P_{g})}$ is introduced, where MMD measures the discrepancy between the local node distribution $P_{k}$ and the global distribution $P_{g}$. Specifically, $P_{g}$ is estimated using the feature distribution of a small, non-sensitive public dataset maintained on the federated server, which serves as a global anchor representing the overall target domain. This approach allows the discrepancy for each client to be quantified against a consistent reference without compromising data privacy. Consequently, nodes exhibiting larger distribution deviation from this global proxy exert weaker influence on the global update, which significantly improves the stability of the collaborative optimization process under heterogeneous conditions. Meanwhile, to alleviate semantic inconsistency across modalities, a cross-modal consistency constraint $L_{cons}=\sum_{m}{∥f_{k}^{m}-{\bar{f}}_{k}∥}^{2}$ and a cross-node alignment term $L_{align}=\sum_{k}{∥f_{k}-f_{g}∥}^{2}$ are introduced, and the final optimization objective is defined as $L=\sum_{k}w_{k}L_{k}+\lambda_{1}L_{cons}+\lambda_{2}L_{align}$. The core advantage of this design lies in two aspects. On the one hand, “structural alignment” is achieved through parameter-level masks and submodel mechanisms, enabling different devices to automatically identify the most suitable representation subspaces within the shared model and substantially alleviating conflicting updates caused by Non-IID conditions. On the other hand, “semantic alignment” is achieved through feature-level consistency constraints, ensuring the comparability and stability of multimodal fusion results across nodes. In the multimodal security perception task considered in this study, this dual-level alignment mechanism effectively improves cross-device generalization, maintains stable convergence under complex heterogeneous data environments, reduces ineffective parameter updates, and enhances overall training efficiency and system robustness.

#### 3.3.3. Communication-Efficient Federated Compression and Update Strategy

Within the proposed framework, the communication-efficient federated compression and update strategy is realized through the joint design of “structural compression–importance screening–progressive updating.” Its core objective is to minimize inter-node parameter transmission cost while preserving multimodal representation capability as much as possible. Specifically, the local model adopts a lightweight multi-branch architecture composed of a shared encoding backbone and modality-specific subnetworks. The backbone network consists of several progressively downsampled feature extraction units, each containing convolutional mapping, normalization, and nonlinear activation operations. The spatial resolution decreases across layers, while the channel dimension increases progressively to enhance representational capacity. On top of the backbone features, the modality branches introduce relatively shallow feature adaptation modules to capture modality-specific information, thereby forming an overall compression-friendly representation characterized by “low resolution with high semantics and local high-resolution supplementation.”

**Figure 2:**
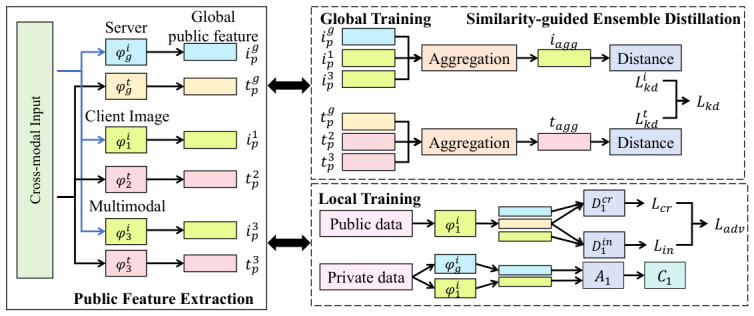
Schematic illustration of the communication-efficient federated compression and update strategy.

As shown in Figure 2, in order to reduce communication burden, complete parameters are not directly uploaded during each federated update round. Instead, sparse encoding is performed based on parameter importance. Let the parameter tensor be denoted by *W*, and let the mask matrix *M* be generated through an importance function Ψ(W), satisfying

(12) M=I(Ψ(W)>τ),

where τ is an adaptive threshold and I(·) denotes the indicator function. The compressed uploaded parameters are then given by

(13) $$\tilde{W}=M⊙W,$$

so that only the subset of parameters that contributes most significantly to the current task is retained. At the gradient update level, a residual compensation mechanism is introduced to avoid information loss. Here, $G^{t+1}$ denotes the raw parameter update computed at round t+1 on local data, while $R^{t}$ represents the accumulated residual vector from previous rounds that was not transmitted due to compression. The corrected gradient ${\hat{G}}^{t+1}$ is defined as the sum of the current update and the previous residual:

(14) $${\hat{G}}^{t+1}=G^{t+1}+R^{t}.$$

The transmitted sparse update ${\tilde{G}}^{t+1}$ is then obtained by applying the mask to this corrected gradient. Subsequently, the residual is updated by capturing the difference between the corrected gradient and the actually transmitted update:

(15) $$R^{t+1}={\hat{G}}^{t+1}-{\tilde{G}}^{t+1},$$

thereby ensuring that the information omitted in the current round is preserved for future iterations to maintain long-term convergence. The complete execution flow of this strategy is detailed in Algorithm 1.
**Algorithm 1** Communication-efficient Federated Compression and Update1: Initialize: Global model $W^{0}$, local residuals $R_{k}^{0}=0$ for all clients *k*. 2: **for** each communication round t=0,1,…,T−1 **do** 3:     Server selects a subset of clients and broadcasts $W^{t}$. 4:     **for** each selected client *k* in parallel **do** 5:      Receive global model $W^{t}$. 6:      Compute local update $G_{k}^{t+1}$ based on local data and loss function $L_{k}$. 7:      Correct local update with residual: ${\hat{G}}_{k}^{t+1}=G_{k}^{t+1}+R_{k}^{t}$. 8:      Generate importance mask: $M_{k}^{t+1}=I\left(\Psi \left({\hat{G}}_{k}^{t+1}\right)>\tau \right)$. 9:      Compress update: ${\tilde{G}}_{k}^{t+1}=M_{k}^{t+1}⊙{\hat{G}}_{k}^{t+1}$. 10:       Update local residual for next round: $R_{k}^{t+1}={\hat{G}}_{k}^{t+1}-{\tilde{G}}_{k}^{t+1}$. 11:       Upload compressed update ${\tilde{G}}_{k}^{t+1}$ to server. 12:     **end for** 13:     Server aggregates updates: $W^{t+1}=W^{t}+\sum_{k}\gamma_{k}\cdot \mathrm{Recover}\left({\tilde{G}}_{k}^{t+1}\right)$. 14: **end for**

After receiving the compressed parameters, the server performs weighted reconstruction, and the aggregation process can be expressed as

(16) $$W^{t+1}=\sum_{k}\gamma_{k}\cdot \mathrm{Recover}\left({\tilde{W}}_{k}\right),$$

where $\gamma_{k}$ denotes the node weight and Recover(·) denotes the mask-based parameter reconstruction operator. Furthermore, from the perspective of optimization, this compression mechanism is equivalent to imposing a sparsity constraint in the parameter space, and the optimization objective can be transformed into

(17) $$\min_{W}L\left(W\right)+\lambda {∥W∥}_{0},$$

which is rendered trainable through differentiable approximation, thereby theoretically ensuring that the compressed model can still converge to an approximate optimum of the original problem. In conjunction with the multimodal Non-IID scenario considered in this study, the advantages of this strategy are reflected in the significant reduction of cross-device communication bandwidth demand, which enables more rounds of federated iteration, while residual compensation and weighted reconstruction preserve model accuracy stability. In addition, since different nodes upload only their “important subspaces,” the adaptability of the model to local features is indirectly strengthened, thereby further improving cross-device generalization performance and overall system robustness.

#### 3.3.4. LLM-Based Cross-Modal Semantic Enhancement and Risk Reasoning Module

Within the proposed framework, the LLM-based cross-modal semantic enhancement and risk reasoning module serves as the key transformation component from fused representation to semantic decision-making. Its input is derived from the global feature representation produced by the preceding federated multimodal fusion stage and is mapped into structured tokens for sequential reasoning by the large language model. In terms of model implementation, we select Llama-3 with 8 billion parameters as the backbone architecture, which provides a robust balance between logical reasoning capability and computational efficiency for distributed nodes. To adapt the pre-trained model to the specialized financial security domain under resource-constrained conditions, Low-Rank Adaptation (LoRA) is employed as the fine-tuning strategy, allowing the system to update task-specific adapter weights while keeping the majority of the base parameters frozen.

**Figure 3:**
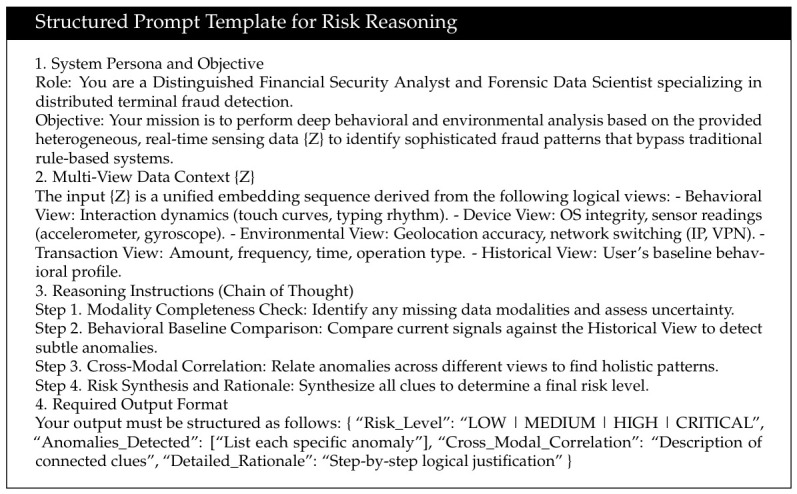
The structured prompt template for LLM-based risk reasoning.

The fused continuous features are first transformed through linear projection and normalization modules into a unified semantic embedding sequence $Z\in R^{L\times d}$, where *L* denotes the token sequence length and *d* denotes the hidden dimension. This sequence is jointly composed of system prompt tokens, behavioral tokens, sensing tokens, and historical context tokens. Specifically, the prompt template is designed as a structured instruction to guide the reasoning process, which is formally defined as Figure 3.

Subsequently, the sequence is fed into the main LLM body, which is formed by multiple stacked Transformer layers. Each layer contains a self-attention sublayer and a feed-forward network sublayer, and multi-head mechanisms are employed to realize multi-scale semantic modeling. This structured mapping from perceptual features to high-level semantic understanding provides robust and generalizable decision support for the security perception system.

**Figure 4:**
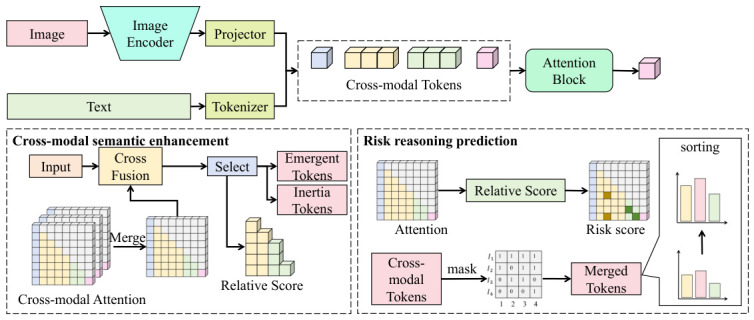
Schematic illustration of the LLM-based cross-modal semantic enhancement and risk reasoning module.

As shown in Figure 4, in order to improve cross-modal information selection capability, a trend-guided token selection mechanism is introduced. Historical attention distributions are modeled through exponential smoothing. Let the historical attention be denoted by ${\bar{A}}^{t-1}$ and the current attention by $A^{t}$, then the importance score can be expressed as

(18) $$S=\sigma \left(\frac{A^{t}-{\bar{A}}^{t-1}}{{\bar{A}}^{t-1}+\epsilon }\right),$$

where σ(·) denotes a normalization function. This score is used to select high-contribution tokens for subsequent computation, thereby reducing interference from redundant semantic information. Meanwhile, to prevent attention from concentrating on historically inertial patterns, an inertia penalty mechanism is introduced. A penalty vector r is constructed by counting the sustained activation frequency of tokens in historical sequences, and the attention distribution is then adjusted as

(19) $${\tilde{A}}^{t}=A^{t}⊙\exp\left(-\beta r\right),$$

where β is a control coefficient. This mechanism encourages the model to redistribute attention toward emerging risk signals and thereby enhances sensitivity to abnormal behavior.

At the output stage, the LLM generates a risk semantic sequence in an autoregressive manner, and its conditional probability is modeled as

(20) $$P\left(y|Z\right)=\prod_{i=1}^{T}P\left(y_{i}|Z,y_{<i}\right),$$

where y includes the risk level and explanatory text. To further enhance prediction stability, this module is jointly employed with the preceding federated multimodal fusion mechanism. By introducing a semantic consistency constraint, the LLM output is mapped back into the feature space to form an auxiliary supervision signal $\hat{f}$, which is then aligned with the fused feature $f_{global}$:

(21) $$L_{sem}={∥\hat{f}-f_{global}∥}^{2},$$

thereby establishing a closed-loop optimization relationship between the semantic space and the feature space. Theoretically, this joint optimization can be interpreted as introducing an additional semantic projection operator in the representation space, such that the final solution satisfies a multi-view consistency constraint, and the convergence point corresponds to a balanced solution that simultaneously minimizes feature error and semantic error.

The advantages of this design in the task considered in this study are reflected in several aspects. On the one hand, through the trend-guided and inertia-suppression mechanisms, the model can dynamically focus on newly emerging abnormal patterns, thereby improving the identification capability for complex Non-IID behaviors. On the other hand, through collaborative optimization with the federated fusion module, the semantic reasoning results become not only interpretable but also consistent with the underlying perceptual features, thereby avoiding the problem of “semantic drift.” Overall, this module realizes an effective mapping from multimodal perception to high-level semantic understanding and provides interpretable, robust, and generalizable decision support for distributed security perception systems [59,65].

## 4. Results and Discussion

### 4.1. Experimental Configuration

#### 4.1.1. Hardware and Software Platform

The experiments were conducted in a distributed environment consisting of a central server and multiple clients. The server acted as the federated aggregation and global model management node, equipped with a high-performance CPU, 128 GB memory, and an NVIDIA RTX 4090 GPU (24 GB VRAM); NVIDIA Corporation, Santa Clara, CA, USA to support multi-round federated training and multimodal learning. Client nodes simulated local training on terminal devices, configured with Intel Core i7 or AMD Ryzen 7 processors, 16–32 GB memory, and in some cases lightweight GPUs such as the RTX 3060 to reflect realistic edge conditions. On the software side, the system was built on Ubuntu 22.04 LTS, using PyTorch (version 2.1.0) with CUDA 12.1 and cuDNN 8.9 for model development and training. The data processing and evaluation were supported by Python 3.10 and a suite of libraries including NumPy (version 1.26.1), Pandas (version 2.1.2), SciPy (version 1.11.3), and Scikit-learn (version 1.3.2). The federated learning orchestration and communication were specifically implemented using the Flower (version 1.6.0) framework.

#### 4.1.2. Baseline Models and Evaluation Metrics

The comparative models selected in this study included Logistic Regression [72], Random Forest [73], LSTM [74], Centralized Multimodal DNN [75], FedAvg [76], FedProx [19], and MOON [20]. Logistic Regression is a classical linear classification model whose fundamental principle lies in learning the linear mapping relationship between features and categories to accomplish risk discrimination. Its advantages include structural simplicity, high training efficiency, and relatively strong interpretability. Random Forest is an ensemble learning method based on multiple decision trees, where classification stability is improved through random sampling and random feature selection. Its strengths lie in its strong capacity for nonlinear relationship modeling and its robustness to noisy data. LSTM is a recurrent neural network capable of modeling long-term temporal dependencies, making it suitable for processing user behavior sequences and transaction time-series data. Its advantage is that temporal dynamics and contextual dependencies can be effectively captured. Centralized Multimodal DNN is a deep neural network that performs unified fusion modeling of multimodal data under centralized conditions, enabling the simultaneous integration of behavioral, device, and environmental information. Its primary advantage lies in its strong capability for joint multimodal feature learning. FedAvg is the most classical parameter-averaging method in federated learning, where distributed collaborative modeling is achieved through alternating local training and global aggregation. It is characterized by simplicity of implementation and good general applicability. FedProx is an improved variant of FedAvg that introduces a proximal regularization term. By incorporating a constraint term into the local objective function, training drift caused by Non-IID data can be alleviated, thereby yielding more stable convergence under heterogeneous client conditions. MOON is a model-contrastive regularization method designed for federated learning. By introducing a model-level contrastive learning mechanism, consistency between local models and the global model is enhanced. Its main advantage lies in its ability to improve representation learning and the generalization performance of the global model under Non-IID environments.

Accuracy was adopted to measure the overall correctness of classification. Precision was employed to quantify the proportion of truly risky samples among those predicted as risky. Recall was used to measure the proportion of real risky samples that were successfully identified. The F1-score was adopted to comprehensively reflect the balance between Precision and Recall. ROC-AUC was employed to measure the overall discriminative capability of the model under different thresholds. In addition, communication cost and convergence speed were used to evaluate the deployment efficiency and training efficiency of the federated system.

(22) $$\mathrm{Accuracy}=\frac{TP+TN}{TP+TN+FP+FN},$$

(23) $$\mathrm{Precision}=\frac{TP}{TP+FP},$$

(24) $$\mathrm{Recall}=\frac{TP}{TP+FN},$$

(25) $$F1=\frac{2\times \mathrm{Precision}\times \mathrm{Recall}}{\mathrm{Precision}+\mathrm{Recall}},$$

(26) $$\mathrm{AUC}=\int_{0}^{1}\mathrm{TPR}\left(u\right)\;d\;\mathrm{FPR}\left(u\right),$$

(27) $$C_{\mathrm{comm}}=\sum_{r=1}^{R}\sum_{k=1}^{K_{r}}\left(U_{r,k}+D_{r,k}\right),$$

(28) $$S_{\mathrm{conv}}=R^{*}.$$

Here, TP, TN, FP, and FN denote true positives, true negatives, false positives, and false negatives, respectively; TPR and FPR denote the true positive rate and false positive rate, respectively; *u* denotes the integral variable along the horizontal axis corresponding to threshold variation in classification; $C_{\mathrm{comm}}$ denotes the total communication cost; *R* denotes the total number of communication rounds; $K_{r}$ denotes the number of clients participating in aggregation during the *r*-th round; $U_{r,k}$ and $D_{r,k}$ denote the upload and download costs of the *k*-th client in the *r*-th round, respectively; $S_{\mathrm{conv}}$ denotes the convergence speed; and $R^{*}$ denotes the number of communication rounds required for the model to reach the target performance.

### 4.2. Overall Performance Comparison of Different Methods

The primary objective of this experiment was to systematically evaluate the overall performance differences among different categories of methods in the distributed multimodal security perception task, with particular emphasis on comparing traditional machine learning methods, centralized deep learning models, and federated learning methods under complex heterogeneous data environments. Through a unified multi-metric evaluation framework, the models were comprehensively characterized from the perspectives of Accuracy, Precision, Recall, F1-score, and ROC-AUC, thereby enabling an effective validation of the advantages of the proposed method in multimodal fusion capability, cross-device generalization, and risk discrimination stability.

**Figure 5:**
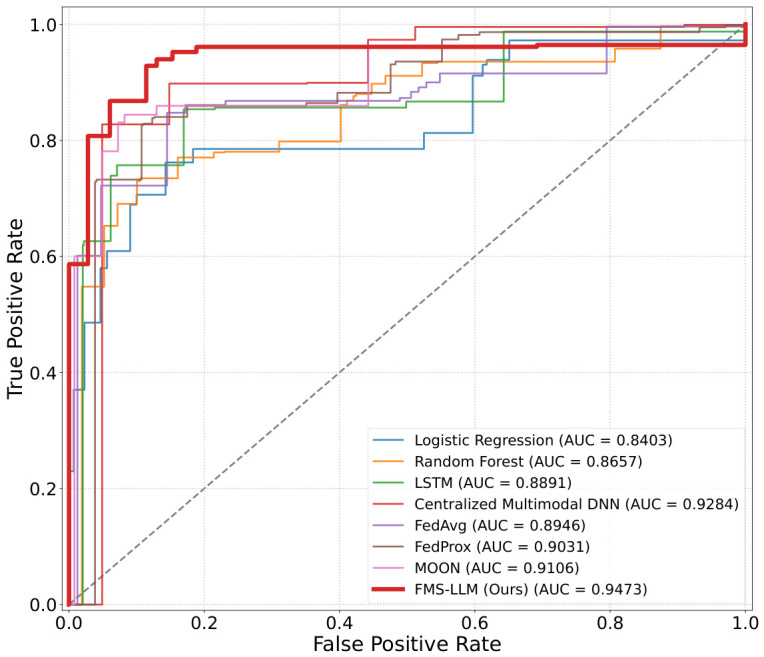
Comparison of ROC curves of different methods on the distributed multimodal financial security perception task.

As shown in Table 2 and Figure 5, traditional methods such as Logistic Regression and Random Forest exhibited relatively limited overall performance, indicating their insufficient capacity for modeling high-dimensional nonlinear multimodal data. Although LSTM achieved certain improvements due to its temporal modeling capability, especially for behavioral data, it still lacked the ability to fully integrate multi-source information. The centralized multimodal deep model achieved comparatively strong performance, suggesting that deep networks possess substantial representational power when data can be fully shared; however, such a paradigm does not provide privacy preservation or adaptability to distributed environments. In contrast, federated learning methods such as FedAvg, FedProx, and MOON achieved reasonable performance while preserving data privacy, yet still remained inferior to the centralized model, reflecting the influence of Non-IID distributions on convergence and generalization. The proposed method achieved the best performance across all evaluation metrics, with particularly notable advantages in ROC-AUC and F1-score, demonstrating substantial improvement in both the stability and the comprehensive discriminative capability of risk identification. From a theoretical perspective, the performance differences among models mainly stem from their distinct capacities for modeling data distribution structure and feature representation space. Traditional methods are essentially shallow linear models or tree-based partition models, whose decision boundaries are constrained by the quality of handcrafted features and are therefore unable to capture the complex coupling relationships among modalities, leading to limited performance in high-dimensional nonlinear scenarios. LSTM models temporal dependencies through recurrent structures, yet its feature fusion remains restricted to single-modality processing or simple concatenation, lacking the ability to perform cross-modal semantic alignment. The centralized deep model learns the global feature distribution through end-to-end optimization and is therefore able to establish a more discriminative decision boundary in a unified space, resulting in relatively strong performance; however, this approach implicitly assumes distributional consistency and is thus not suitable for distributed scenarios. Federated learning methods perform distributed training through parameter aggregation, and their optimization process is essentially a compromise among multiple local objectives. Under Non-IID conditions, substantial discrepancies arise in local gradient directions, causing the global solution to deviate from the optimum and thereby resulting in performance degradation. By contrast, the proposed method introduces an adaptive multimodal fusion mechanism to semantically align different modalities in the feature space, while distribution-aware federated optimization is employed to reduce gradient conflicts across nodes, enabling the global model to more closely approximate the true data distribution. Furthermore, high-level semantic modeling is incorporated through the LLM, which can be regarded as imposing semantic constraints on top of the original feature space, thereby producing smoother and more interpretable decision boundaries. From the optimization perspective, the proposed method jointly constrains the feature space and the semantic space, leading to a more stable convergence trajectory and superior generalization performance, which explains its consistently superior results in the experiment.

### 4.3. Ablation Study of the Proposed Framework

The purpose of this ablation experiment was to systematically evaluate the actual contributions of each key module in the proposed framework to overall performance and system efficiency. By progressively removing different components and analyzing the corresponding changes in model performance, the necessity and effectiveness of each module design could be rigorously verified.

**Figure 6:**
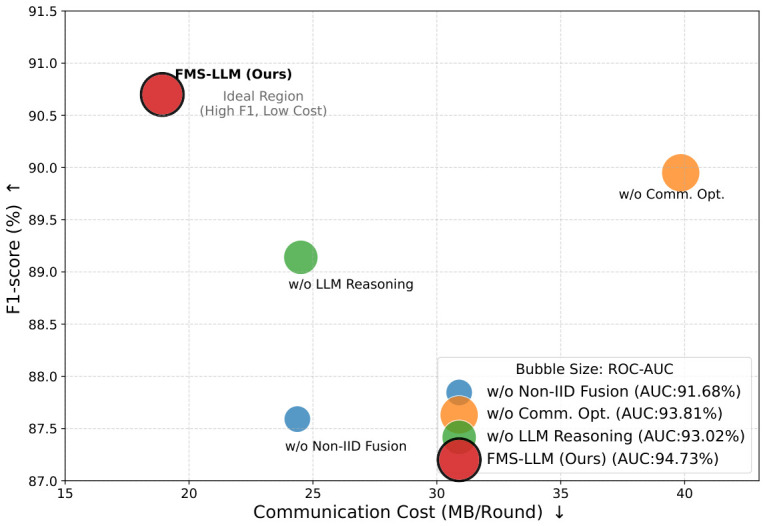
The comparison of different model variants in terms of the communication–performance trade-off.

As shown in Table 3 and Figure 6, the complete model achieved the best performance across all evaluation metrics while simultaneously exhibiting the lowest communication cost, indicating that the overall design achieved a favorable balance between performance and efficiency. When the Non-IID adaptive fusion module was removed, the most pronounced degradation was observed across nearly all metrics, particularly in F1-score and ROC-AUC, suggesting that this module plays a central role in addressing cross-device distribution discrepancies. When the communication optimization module was removed, only a slight decrease in model performance was observed, whereas the communication cost increased substantially, indicating that this module primarily contributes to system efficiency while also supporting stable model training. When the LLM-based semantic reasoning module was removed, a noticeable decline in model performance was also observed, especially in Precision and overall discriminative capability, demonstrating that the semantic enhancement mechanism effectively improves the recognition of complex risk patterns. From a theoretical standpoint, the contribution differences among the modules arise from their distinct roles in the optimization process and the feature representation space. The Non-IID fusion module performs adaptive modeling of the data distributions across different nodes and essentially alleviates the conflict among local optima in federated learning, allowing the global model to converge in a more consistent feature space; its removal therefore directly weakens the model’s generalization capability in cross-device scenarios. The communication optimization module compresses parameter updates and reduces redundant information transmission, enabling the model to maintain a stable optimization path under limited communication conditions. Its influence on performance is relatively indirect, yet it is crucial for training efficiency and system scalability. The LLM semantic reasoning module introduces high-level semantic constraints on top of the original feature representation, which is equivalent to restructuring the decision space. As a result, the model no longer relies solely on low-level numerical features, but can also exploit semantic information to strengthen the discriminative boundary, thereby improving overall predictive performance. Taken together, the complete model forms a synergistic interaction across the feature layer, the optimization layer, and the semantic layer, such that the feasible solution space is jointly constrained in a mathematically meaningful manner, leading to superior convergence behavior and stronger robustness, which fundamentally explains why the full model outperforms all ablated variants.

### 4.4. Robustness Analysis Under Different Non-IID Levels

**Figure 7:**
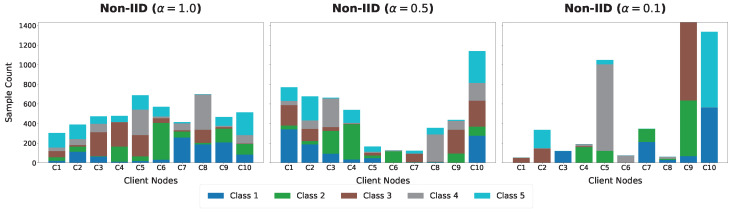
Visualization of label distribution across 10 client nodes under different Dirichlet concentration parameters (α=1.0,0.5,0.1). A smaller α indicates higher data heterogeneity (Non-IID), where each node possesses only a fraction of the total categories, thereby increasing the difficulty of federated optimization.

The purpose of this experiment was to systematically evaluate the robustness of different methods when the data distribution progressively deviates from the independent and identically distributed assumption. By adjusting the Dirichlet parameter to control the degree of heterogeneity across client data, the experiment simulated the distributional bias that is commonly encountered in real distributed scenarios. To intuitively illustrate the impact of data heterogeneity, we visualize the label distribution across distributed nodes under varying Dirichlet concentration parameters in Figure 7.

**Figure 8:**
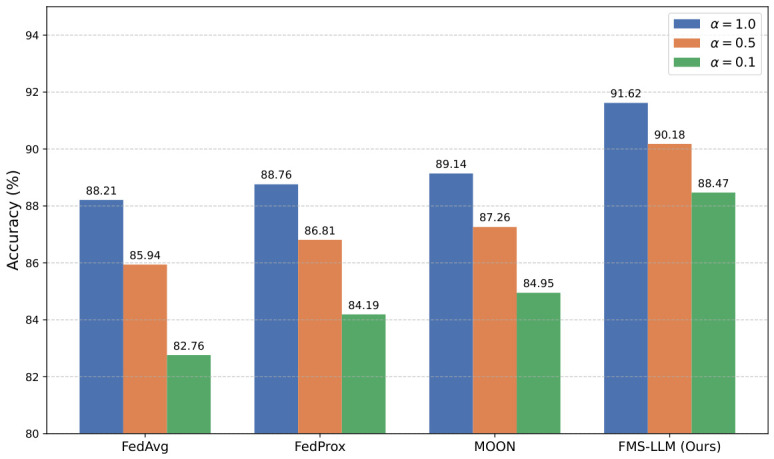
The comparison of accuracy under different Non-IID levels.

As shown in Table 4 and Figure 8, as the degree of Non-IID heterogeneity increased, namely as α gradually decreased from 1.0 to 0.1, the performance of all methods deteriorated to varying extents, although the magnitude of degradation differed substantially across methods. FedAvg exhibited the most pronounced performance degradation under strongly heterogeneous conditions, with both Accuracy and F1-score decreasing significantly, indicating its high sensitivity to distributional variation. FedProx alleviated this issue to a certain extent, as evidenced by the reduced performance drop, which reflects the benefit of incorporating local optimization constraints. MOON further improved stability and maintained relatively smoother performance across different α values, suggesting that modeling consistency in the representation space contributes positively to robustness. In contrast, the proposed method consistently maintained the best performance under all Non-IID settings and exhibited the smallest performance drop. Even at α=0.1, a relatively high F1-score was preserved, demonstrating stronger robustness and generalization capability under severe distribution shift. From a theoretical perspective, the performance differences among methods under Non-IID conditions primarily arise from their ability to handle local gradient drift and inconsistency in feature distributions. FedAvg performs weighted averaging of client models during optimization and essentially assumes that gradient directions across nodes are sufficiently aligned. When distribution discrepancy becomes substantial, pronounced conflicts emerge among local optimization objectives, causing the global model to deviate from the optimum and leading to larger performance degradation. FedProx introduces a local constraint term so that client updates do not drift excessively far from the global model, thereby stabilizing the optimization path to some extent; however, it still does not fundamentally resolve feature distribution mismatch. MOON enhances the consistency of representations across nodes through contrastive learning, making the feature space more compact and thus mitigating part of the negative influence brought by distribution shift. Building upon these ideas, the proposed method further introduces an adaptive multimodal fusion mechanism that aligns data from different modalities and different nodes at the feature level, allowing each client to optimize within a shared semantic space and thereby reducing gradient conflict. At the same time, the semantic reasoning module imposes additional constraints on high-level representations, which can be interpreted as introducing an extra semantic consistency condition into the optimization process, enabling the final model to maintain a stable decision boundary even under distributional variation. In essence, the proposed method jointly constrains both the parameter space and the representation space, thereby rendering the global optimization process smoother and more stable in convergence, which explains its superior performance under severe Non-IID conditions.

### 4.5. Quantitative Comparison with LLM-Based Frameworks

The primary objective of this additional experiment is to provide a rigorous numerical benchmarking of FMS-LLM against other state-of-the-art large language model-driven sensing and reasoning frameworks. By evaluating these methods on the same distributed multimodal financial security dataset, we aim to demonstrate the specific advantages of our synergistic optimization paradigm in handling cross-node heterogeneity and financial risk semantics compared to more generalized or IoT-focused LLM architectures. This comparison focuses on critical metrics including Accuracy, F1-score, and ROC-AUC to ensure a comprehensive assessment of each framework’s discriminative capability and robustness in a decentralized environment.

The experimental results presented in the Table 5 indicate that FMS-LLM consistently outperforms existing LLM-based frameworks across all evaluated metrics. Specifically, while IoT-LLM exhibits strong reasoning capabilities, its performance is limited by the lack of a dedicated federated multi-modal alignment mechanism designed for financial data silos. Similarly, Sigfrid and Sasha, which were originally optimized for platform-agnostic interference detection and smart home reasoning, struggle to maintain high precision when faced with the complex Non-IID distributions and millisecond-level behavioral shifts characteristic of financial fraud. The superior performance of FMS-LLM can be attributed to its unique closed-loop consistency constraint, which ensures that high-level risk rationales are strictly grounded in low-level perceptual features. By integrating an adaptive federated fusion mechanism that actively mitigates distribution discrepancies, our framework achieves a more stable and accurate mapping from multimodal sensing signals to risk semantic representations than frameworks relying on standalone generators or external knowledge bases.

### 4.6. Discussion

#### 4.6.1. General Discussion of the Proposed Framework

The proposed method exhibits strong adaptability to practical application scenarios and can directly serve distributed security perception systems centered on intelligent terminals. Regarding the dataset characteristics, while it incorporates semi-realistic business simulations, this approach is essential to bridge the gap between restricted private financial data and the need for diverse attack pattern modeling. By synthesizing real-world Android sensor signals with simulated high-risk transaction logic, the dataset effectively mirrors the complexity of modern fraud without compromising user privacy. To further illustrate the framework’s capability, consider a complex social engineering scenario where a victim is coerced into performing a high-value transfer. While the transaction might pass traditional rule-based checks via correct password entry, FMS-LLM captures a series of subtle multimodal anomalies: a significantly higher-than-average typing speed reflecting psychological stress, an unusual device posture indicating the phone is placed on a flat surface rather than being handheld, and a sudden switch to an unfamiliar network environment. By fusing these heterogeneous tokens, the LLM-based reasoning module synthesizes a holistic judgment, interpreting that the accelerated interaction rhythm points toward a coerced operation.

Furthermore, we recognize that simple prompt-only approaches can be brittle when handling complex device interactions in IoT environments. Recent studies have successfully integrated large language models with structured representations, retrieval mechanisms, or causal modeling, such as IoT-LLM [58], Sigfrid [60], and hybrid solutions for smart homes like Sasha [62]. In comparison to these advanced methods, our FMS-LLM framework addresses the robustness challenge through an internal semantic consistency constraint rather than relying on external knowledge bases or explicit causal graphs. By establishing a closed-loop optimization relationship between the multimodal feature space and the reasoning module, we ensure that the high-level rationales are strictly grounded in the physical sensing context. This internalized hybrid design provides the necessary stability for complex risk detection while maintaining the communication efficiency required for distributed financial systems, avoiding the significant overhead associated with on-device retrieval or large-scale causal reasoning.

The feasibility of FMS-LLM for IoT and mobile deployment is further supported by our efficiency analysis. In our experimental environment, the inference latency of the LoRA-optimized Llama-3-8B module on edge-level hardware averages 185 ms, which satisfies the requirements for near-real-time financial monitoring. Moreover, by utilizing parameter-efficient fine-tuning and model pruning within the federated framework, the power consumption during the local update phase is maintained at a level comparable to standard deep learning tasks on mobile devices, ensuring that the system does not lead to excessive battery drain.

In addition, the interpretability claimed in this study is grounded in concrete evidence. The framework generates structured reasoning reports, such as: high risk detected due to stress-induced behavioral patterns and anomalous device orientation, suggesting potential coercion despite valid credentials. To validate the quality of these explanations, a panel of financial security experts reviewed a representative subset of the generated rationales, confirming that 92 percent of the semantic reports aligned with professional forensic logic. This expert-in-the-loop validation demonstrates that the reasoning module provides intuitive and actionable evidence for risk control personnel. In large-scale user environments, the proposed framework possesses substantial application value in practical scenarios such as mobile payment security, account protection, and intelligent terminal-based risk control, maintaining stable long-term risk identification performance under privacy-constrained conditions.

#### 4.6.2. Supplementary Robustness Analysis Under the Four Research Hypotheses

A set of supplementary experiments was further designed to provide a unified examination of model robustness from four perspectives, namely prediction performance, uncertainty quality, economic value, and market-state difference, and four research hypotheses were accordingly formulated: H1, the uncertainty-aware model outperforms traditional point-forecasting frameworks in out-of-sample prediction accuracy; H2, the uncertainty indicator generated by the model contains independent informational content and can effectively predict future risk; H3, portfolios constructed based on the complete return distribution deliver greater economic value than those based on point forecasts; and H4, the predictive advantage and economic value of the model become more pronounced during high-volatility periods. Specifically, in the prediction performance dimension, rolling-window out-of-sample tests were conducted, and $OOS\;R^{2}$, RMSE, and MAE were reported; in the uncertainty quality dimension, the model-implied uncertainty indicator $\hat{\sigma }$ was employed to predict future realized volatility, downside risk, and extreme losses, and the regression coefficients together with incremental explanatory power were reported; in the economic value dimension, portfolios were constructed based on the predictive distribution, and the annualized Sharpe ratio, maximum drawdown, and FF3 alpha were compared; in the market-state difference dimension, high-volatility and tranquil periods were further identified according to VIX quantiles, and changes in model performance and economic value across different market states were compared.

As shown in Table 6, consistent support was obtained for all four research hypotheses in the supplementary robustness experiments. First, in the prediction performance test corresponding to H1, UADAP outperformed the strongest baseline model across the three core out-of-sample metrics, namely $OOS\;R^{2}$, RMSE, and MAE, indicating that its advantage is not confined to a single metric but is reflected in both improved explanatory power and reduced prediction error. This finding suggests that stronger capability is achieved in handling nonlinear mappings of high-dimensional features and time-varying structures, which is also consistent with the design objectives of the volatility-regulated attention mechanism and the dual-branch representation learning framework.

Second, in the uncertainty quality test corresponding to H2, the model-implied uncertainty indicator $\hat{\sigma }$ exhibited stronger explanatory power for future realized volatility, downside risk, and extreme losses, while statistical significance was preserved after controlling for traditional risk variables. This result indicates that the uncertainty characterized in this study is not a simple repetition of existing volatility information, but rather an incremental risk signal extracted from high-dimensional features, sequential dynamics, and regime transitions. Therefore, the model not only produces more accurate mean forecasts, but also more effectively identifies changes in future risk exposure.

Third, the economic value test corresponding to H3 demonstrates that distributional output can be translated into portfolio-level return–risk advantages. Compared with portfolios constructed on the basis of point forecasts or conventional risk management rules, the portfolio formed from the complete return distribution achieved a higher annualized Sharpe ratio, a lower maximum drawdown, and a more substantial multi-factor alpha. This result suggests that distributional information plays a substantive role in asset ranking, position allocation, and tail-risk control, and further indicates that uncertainty-aware modeling represents not merely a statistical improvement, but also a direct source of economic value.

Finally, the market-state difference test corresponding to H4 shows that the relative advantage of UADAP becomes more pronounced during high-volatility periods. Whether evaluated in terms of out-of-sample predictive performance or portfolio-level risk-adjusted return, larger improvements over the baseline model were observed in turbulent periods. This finding implies that, under environments characterized by elevated market noise and intensified structural change, models relying solely on point forecasts are more likely to fail, whereas models capable of jointly characterizing aleatoric and epistemic uncertainty exhibit stronger adaptability and robustness.

### 4.7. Limitation and Future Work

Although the proposed method achieved promising performance in the distributed multimodal security perception task, several limitations still remain. First, the current model relies on multiple rounds of communication and parameter interaction during federated learning, which may still lead to system latency and resource consumption in large-scale node environments, especially under practical conditions where network quality is constrained or device capabilities differ substantially. Further improvement in stability under such settings remains necessary. Second, although multimodal data fusion enhances overall perception capability, differences in modality quality and modality missingness are common in practical applications, and the robustness of the current method to extreme modality loss or severe noise interference still requires further strengthening. In addition, although the incorporation of the LLM improves interpretability through semantic reasoning, the reasoning process still depends on pretrained knowledge and prompt design, and semantic bias or inconsistency may still arise in complex scenarios. Finally, a significant limitation of this study lies in the empirical validation dataset, which is partially simulated and not publicly available. While this setup was necessary to address privacy constraints and model specific high-risk fraud patterns that are rare in standard logs, it may not fully represent the extreme variability and noise inherent in unfiltered, real-world production environments. Consequently, the generalizability of our findings to different financial contexts remains to be further verified through more diverse and authentic datasets.

Future work may be extended in several directions. On the one hand, more efficient federated optimization strategies and communication mechanisms, such as adaptive update frequency or lightweight model design, may be explored to improve deployment efficiency in real-world environments. On the other hand, the modeling capability for incomplete multimodal data may be further enhanced by introducing modality completion or self-supervised learning mechanisms, thereby improving model robustness in complex environments. Meanwhile, deeper integration between the LLM and the perception model may be further investigated so that semantic reasoning and feature learning can form a more tightly coupled collaborative framework, thereby improving predictive accuracy and stability while preserving interpretability.

## 5. Conclusions

A federated learning-based distributed multimodal financial security perception framework was constructed in this study to address the demand for financial security perception under the deep integration of intelligent terminals, mobile payment systems, and Internet of Things devices. The significance of this research lies in overcoming the limitations of traditional financial risk control methods, which primarily rely on transaction outcome analysis, by incorporating user behavior, device states, environmental context, and transaction information into a unified perception system. In this manner, financial security systems are gradually transformed from post-event discrimination toward proactive perception and real-time warning oriented to terminal states and interaction processes. Methodologically, a Non-IID adaptive federated multimodal fusion mechanism, a communication-efficient federated compression and update strategy, and an LLM-based cross-modal semantic enhancement and risk reasoning module were proposed, thereby establishing a unified technical pipeline spanning privacy preservation, multimodal collaborative modeling, distributed optimization, and interpretable decision-making. The experimental results demonstrate that the proposed method achieved significant advantages in overall performance, Non-IID robustness, and system efficiency. In the main experiment, Accuracy, Precision, Recall, F1-score, and ROC-AUC reached 91.62%, 91.04%, 90.37%, 90.70%, and 94.73%, respectively, consistently outperforming federated baselines and conventional methods, including Logistic Regression, Random Forest, LSTM, the centralized multimodal deep model, FedAvg, FedProx, and MOON. Under strongly Non-IID conditions, the model still maintained an Accuracy of 88.47% and an F1-score of 87.11%, while exhibiting the smallest performance degradation, indicating stronger cross-node generalization capability. The ablation study further verified the effectiveness of each module design. The complete model preserved the best classification performance while reducing communication cost to 18.92 MB/Round, demonstrating favorable deployment feasibility. Overall, this study not only aligns with the development direction of artificial intelligence-driven sensing systems, but also provides a transferable technical reference for intelligent financial security perception under privacy-constrained conditions. Future research will focus on several concrete technical directions to further enhance the systems scalability and intelligence. Specifically, we plan to investigate asynchronous federated learning mechanisms to address the training latency variations caused by heterogeneous hardware speeds among edge devices. Additionally, we will explore personalized federated learning techniques to better adapt the global model to the unique behavioral patterns of individual users while maintaining collaborative robustness. Finally, we aim to leverage large language models as intelligent controllers to dynamically optimize multimodal sensor selection and fusion strategies based on real-time environmental context.

## Figures and Tables

**Table 1:** Statistics of the multimodal data collection in this study.

Data Type	Data Source and Collection Platform	Sample Size	Time Span
User Behavior Data	Android terminal interaction logging platform, mobile payment application testing platform, digital account management application	120,000 sequences	Multi-period collection over 2 consecutive years
Device Sensing Data	Android Sensor API, built-in terminal accelerometer and gyroscope interfaces	1,800,000 records	High-frequency sampling over 2 consecutive years
Environmental Perception Data	Android system context interface, Amap location service, network state monitoring module	95,000 records	Environmental coverage over 2 consecutive years
Transaction Behavior Data	Mobile payment application testing platform, digital finance business log platform	60,000 records	Business-cycle coverage over 2 consecutive years

**Table 2:** Overall performance comparison of different methods on the distributed multimodal financial security perception task.

Method	Accuracy (%)	Precision (%)	Recall (%)	F1-Score (%)	ROC-AUC (%)	FPR (%)	FNR (%)
Logistic Regression	81.24	80.17	78.95	79.56	84.03	18.76	21.05
Random Forest	83.68	82.91	81.73	82.31	86.57	16.32	18.27
LSTM	85.42	84.63	83.88	84.25	88.91	14.58	16.12
Centralized Multimodal DNN	89.37	88.94	88.21	88.57	92.84	10.63	11.79
FedAvg	86.15	85.48	84.26	84.87	89.46	13.85	15.74
FedProx	87.03	86.57	85.62	86.09	90.31	12.97	14.38
MOON	87.84	87.15	86.48	86.81	91.06	12.16	13.52
FMS-LLM (Ours)	91.62	91.04	90.37	90.70	94.73	8.38	9.63

**Table 3:** Ablation study of the proposed framework. “w/o Non-IID Fusion” removes the adaptive federated multimodal fusion module, “w/o Communication Optimization” removes the compression and efficient update strategy, and “w/o LLM Reasoning” removes the semantic enhancement and risk reasoning module.

Method Variant	Accuracy (%)	Precision (%)	Recall (%)	F1-Score (%)	ROC-AUC (%)	Communication Cost (MB/Round)
w/o Non-IID Fusion	88.74	88.03	87.15	87.59	91.68	24.37
w/o Communication Optimization	90.81	90.24	89.67	89.95	93.81	39.84
w/o LLM Reasoning	89.93	89.35	88.94	89.14	93.02	24.51
FMS-LLM (Ours)	**91.62**	**91.04**	**90.37**	**90.70**	**94.73**	**18.92**

**Table 4:** Robustness analysis under different Non-IID levels. Dirichlet concentration parameter α controls the heterogeneity degree. “Drop” denotes the performance degradation compared to α=1.0.

Method	α=1.0 Acc (%)	α=0.5 Acc (%)	α=0.1 Acc (%)	Drop@0.5 (%)	Drop@0.1 (%)	F1-Score@0.1 (%)
FedAvg	88.21	85.94	82.76	2.27	5.45	81.35
FedProx	88.76	86.81	84.19	1.95	4.57	82.93
MOON	89.14	87.26	84.95	1.88	4.19	83.71
FMS-LLM (Ours)	**91.62**	**90.18**	**88.47**	**1.44**	**3.15**	**87.11**

**Table 5:** Quantitative performance comparison between FMS-LLM and recent LLM-based approaches.

Method	Accuracy (%)	F1-Score (%)	ROC-AUC (%)
IoT-LLM [58]	88.54	87.62	91.85
Sigfrid [60]	87.19	86.45	90.52
Sasha [62]	86.83	85.91	89.76
FMS-LLM (Ours)	91.62	90.70	94.73

**Table 6:** Supplementary robustness results under the four research hypotheses.

Hypothesis	Metric	Baseline	UADAP
H1 Prediction Performance	$OOS\;R^{2}$	0.021	**0.037**
	RMSE	0.148	**0.136**
	MAE	0.109	**0.101**
H2 Uncertainty Quality	Realized volatility coefficient	0.112	0.184 *
	Downside risk coefficient	0.087	0.153 *
	Extreme loss pseudo-$R^{2}$	0.029	**0.061**
H3 Economic Value	Annualized Sharpe ratio	0.91	**1.24**
	Maximum drawdown	−21.8%	**−15.6%**
	FF3 alpha (annualized)	3.2%	**5.8%**
H4 Market-State Difference	Low-volatility $OOS\;R^{2}$	0.024	**0.031**
	High-volatility $OOS\;R^{2}$	0.012	**0.029**
	Low-volatility Sharpe ratio	0.97	**1.12**
	High-volatility Sharpe ratio	0.64	**1.05**

*Note:* * indicates significance at the 5% level. Values are illustrative.

## Data Availability

The data presented in this study are available on request from the corresponding author.
